# Weak and Habitat-Dependent Effects of Nutrient Pollution on Macrofaunal Communities of Southeast Australian Estuaries

**DOI:** 10.1371/journal.pone.0065706

**Published:** 2013-06-17

**Authors:** Andrea Nicastro, Melanie J. Bishop

**Affiliations:** Department of Biological Sciences, Macquarie University, North Ryde NSW, Australia; The Australian National University, Australia

## Abstract

Among the impacts of coastal settlements to estuaries, nutrient pollution is often singled out as a leading cause of modification to the ecological communities of soft sediments. Through sampling of 48 sites, distributed among 16 estuaries of New South Wales, Australia, we tested the hypotheses that (1) anthropogenic nutrient loads would be a better predictor of macrofaunal communities than estuarine geomorphology or local sediment characteristics; and (2) local environmental context, as determined largely by sediment characteristics, would modify the relationship between nutrient loading and community composition. Contrary to the hypothesis, multivariate multiple regression analyses revealed that sediment grain size was the best predictor of macrofaunal assemblage composition. When samples were stratified according to median grain size, relationships between faunal communities and nitrogen loading and latitude emerged, but only among estuaries with sandier sediments. In these estuaries, capitellid and nereid polychaetes and chironomid larvae were the taxa that showed the strongest correlations with nutrient loading. Overall, this study failed to provide evidence of a differential relationship between diffuse nutrient enrichment and benthic macrofauna across a gradient of 7° of latitude and 4°C temperature. Nevertheless, as human population growth continues to place increasing pressure on southeast Australian estuaries, manipulative field studies examining when and where nutrient loading will lead to significant changes in estuarine community structure are needed.

## Introduction

The sediment-dwelling invertebrates of estuarine and coastal environments provide important ecosystem services. Suspension feeders remove particles and pollutants from the water, helping to improve clarity [1], [2], [3]. Deposit feeders mix sediments as they feed, increasing the oxygen content of the sediment, vertically transporting sediment particles, and altering sediment stability [4], [5]. Collectively, the sediment-dwelling invertebrates serve as important prey resources for commercially and recreationally important fisheries and, in the intertidal, to migratory shorebirds [6], [7].

Due to the importance of sediment-dwelling communities, a large number of studies have sought to determine those factors that influence their density and diversity. These have shown that at small scales of centimetres to meters, sediment granulometry, sediment organics and flow can influence invertebrate communities [8], [9], [10]. At larger scales, climate (e.g. temperature and for coastal systems, rainfall), geomorphic setting and nutrient loading can be important determinants of community structure [11], [12]. Many of the studies have focused on individual factors, utilizing small-scale aquarium or field experiments to ascertain cause-effect relationships [13], [14]. As human activities increasingly modify coastal and marine environments, large scale field surveys which examine how multiple of these factors cumulatively relate to invertebrate communities are needed to enable appropriate management strategies for the environmental stressors to be developed.

Within temperate estuaries, anthropogenic nutrient enrichment is broadly regarded as one of the greatest modifiers of sediment-dwelling communities, and their dependent ecosystems [15], [16]. Urbanization, deforestation, and agriculture, can lead to diffuse, catchment-scale enhancement of nutrient loading by adding nutrients to the system or by removing terrestrial nutrient stores [17], [18]. Point source discharges, such as from sewage treatment plants, can locally enhance nutrient availability. Point sources provide a continuous and localized source of nutrients, whereas diffuse sources are affected by the freshwater input and rainfall [19], [20], [21]. Where nitrogen (N) and/or phosphorous (P) limits primary production [22], moderate enhancement of the limiting nutrient can stimulate the growth of planktonic and benthic plants and, in turn, the productivity of higher trophic levels [23], [24], [25]. High loadings of nutrients can, however, lead to excess organic matter production (eutrophication), hypoxia or anoxia of bottom sediments [26] and death of benthic organisms [27], [28].

Both local and broad-scale environmental conditions might modify the impact of nutrients on benthic communities. At large scales, climatic setting and estuarine geomorphology might influence whether estuarine waters stratify or not, and hence whether bottom-waters can be reoxygenated following bacterial decomposition of excess primary production, stimulated by nutrient enrichment [29], [28]. The flushing time of an estuary might influence the residence time of nutrients in the estuary, and hence their impact [30]. Further, sediment characteristics might determine the background organic enrichment of the system [31], and hence how close it is to tipping points in community structure that might be approached or exceeded by anthropogenic nutrient enrichment.

Here we conduct sampling across 16 estuaries of New South Wales, Australia, spanning 7° of latitude, to test the hypotheses that: (1) nutrient enrichment will explain more variation in macrofaunal community composition than other environmental variables, including sediment characteristics, estuarine geomorphology and latitude; but (2) the relationship between nutrient enrichment and community composition will be modified by local environmental variables.

## Materials and Methods

### 1. Study Sites and Sampling Design

**Figure 1:**
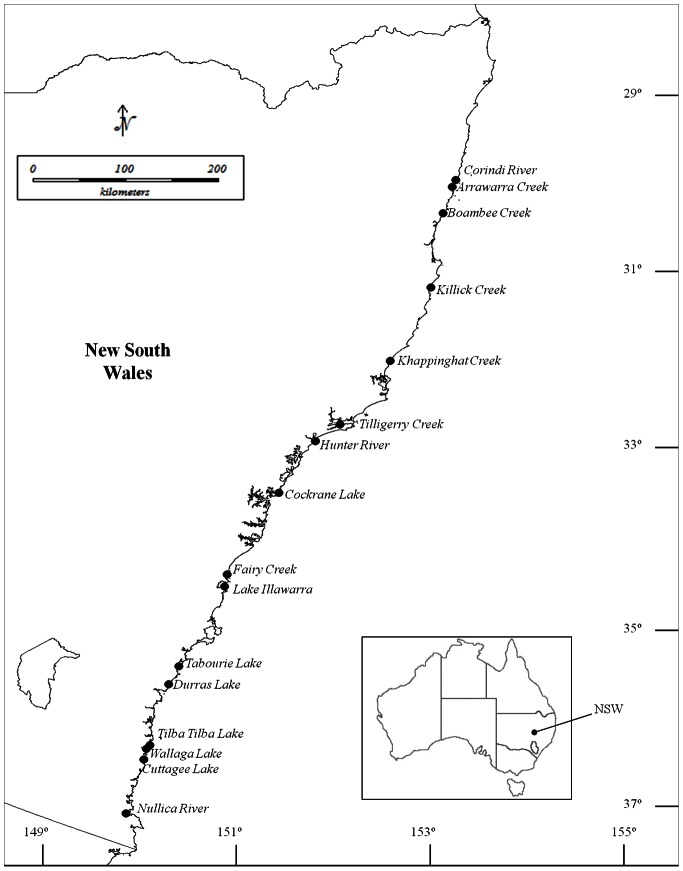
Map showing the location of surveyed estuaries along the coastline of New South Wales (NSW; Australia).

To assess how the local environmental context modulates the effects of nutrient loading on the abundance and diversity of macrobenthic invertebrates, we sampled 16 estuaries along the coast of New South Wales, Australia (Table 1, Fig. 1). Estuaries were chosen along a stretch of coast spanning 7° in latitude, corresponding to a difference in mean annual sea surface temperature of about 4°C [32]. So as to enable a reasonable spread of estuaries along the coastline, the stretch of coast was subdivided into four regions of similar size, within each of which we randomly selected two replicate estuaries receiving a total nitrogen (TN) loading similar to the undisturbed, pre-European settlement levels (ratio of TN loading pre-European settlement to present, <2) and two estuaries that had been subjected to significant anthropogenic nutrient loading (ratio of TN loading pre-European settlement to present, >2.5). The ratios of present day to pre-European TN loading were obtained from the NSW Office of Environment and Heritage [33]. The pre-European TN loading was modelled based on the present TN loading, the spatial extent and typology of human activities that the estuary catchment is presently undergoing and the geomorphological attributes of each estuary (e.g. estuary and catchment area, flushing time; [33]).

**Table 1:** Physical and chemical attributes and mean values of sediment characteristics (mean ± SE, n = 6 for median grain size, sorting and silt/clay content, n = 12 for organic matter [OM] content) for the 16 estuaries surveyed.

Estuary	Lat	Long	Estuaryarea (km^2^)^a^	Catchmentarea (km^2^)^a^	% disturbed catchment area^a^	Actual TN flux (mg m^−2^ d^−1^)^a^	Flushingtime (d^−1^)^a^	TN ratio^a^	Median grainsize (µm)	Sediment sorting (µm)	% Silt/Clay	% OM
Corindi River	29° 59′	153° 14′	1.9	148.3	19.3	53	5.0	1.5	151.4±17.7	310.1±79.2	21.5±6.1	2.1±0.4
Arrawarra Creek	30° 04′	153° 12′	0.1	18.0	14.9	117	3.6	2.5	171.5±1.8	68.5±6.0	1.0±0.2	1.3±0.2
Boambee Creek	30° 21′	153° 06′	1.0	62.2	49.5	237	2.4	10.5	172.6±0.7	210.2±96.3	5.7±2.8	1.5±0.1
Killick Creek	31° 11′	152° 59′	0.3	8.2	30.7	15	56.1	1.9	200.9±6.4	97.2±10.6	0.9±0.2	0.7±0.1
Khappinghat Creek	32° 01′	152° 34′	1.2	91.9	28.4	47	12.2	1.9	252.7±12.6	414.9±75.2	4.5±0.5	3.6±0.5
Tilligerry Creek	32° 44′	152° 03′	134.4	135.2	30.4	1	36.2	3.8	244.0±5.6	119.7±5.0	1.6±0.2	0.6±0.1
Hunter River	32° 53′	151° 48′	47.0	21414.0	61.2	171	5.1	2.6	179.6±70.4	301.0±98.8	30.2±11.6	4.3±1.0
Cockrane Lake	33° 29′	151° 26′	0.3	7.2	38.6	13	31.5	2.0	113.9±72.1	301.4±45.5	46.9±13.8	10.2±4.0
Fairy Creek	34° 24′	150° 54′	0.1	20.8	75.1	487	1.2	3.2	329.0±4.3	140.8±15.1	1.6±0.5	1.1±0.3
Lake Illawarra	34° 32′	150° 53′	35.8	274.3	59.3	8	126.7	3.2	291.8±18.0	212.4±24.0	2.7±1.1	1.3±0.1
Tabourie Lake	35° 27′	150° 25′	1.5	47.6	15.4	11	13.3	1.4	314.8±1.0	112.8±4.5	0.9±0.1	0.6±0.1
Durras Lake	35° 39′	150° 18′	3.8	62.2	6.2	4	102.8	1.3	263. 9±12.6	218.9±55.4	2.7±0.7	2.2±0.3
Tilba Tilba Lake	36° 20′	150° 07′	1.2	18.3	72.4	9	69.6	2.5	325.0±11.0	117.3±7.1	1.2±0.3	0.5±0.1
Wallaga Lake	36° 22′	150° 05′	9.3	273.1	37.4	21	97.4	2.3	392.6±3.7	405.0±124.0	4.1±1.8	2.7±1.6
Cuttagee Lake	36° 29′	150° 03′	1.4	54.5	4.8	17	40.0	1.3	336.2±8.0	126.6±6.4	1.4±0.1	0.8±0.1
Nullica River	37° 06′	149° 52′	0.3	55.1	4.7	40	8.3	1.1	205.6±28.5	135.1±40.0	3.4±1.6	2.3±0.7

Abbreviations: TN flux = flux of total nitrogen, TN ratio = ratio of total nitrogen loading pre-European settlement to present.

a Data from Roper et al. [33].

### 2. Macrofauna and Sediment Properties Methods

Within each of the estuaries, we collected samples of macrofauna and sediment from three 100 m^2^ intertidal sites (∼1.3 m mean tidal range), situated 50–100 metres apart. All sites were fully marine (salinity ranging from 30 to 35 ‰) and sampling was done within 14 days during low tides in late spring (November 2009). Seven replicate sediment cores (10 cm in diameter and 15 cm deep) were randomly collected from unvegetated sediment at each site for faunal analysis. The number of macrofaunal samples was chosen following results from a pilot study in which the species accumulation plot (PRIMER v6.0) reached a plateau after six replicate cores and following previous studies in New South Wales estuaries that have suggested that this level of replication is sufficient to detect treatment effects of manipulations of organic enrichment [34], [35]. Four sediment cores (3 cm in diameter and 10 cm deep) were collected for analysis of organic matter content and sediment size composition. Upon collection, samples for faunal analysis were refrigerated and sieved through a 0.5 mm mesh within 72 hours to remove fine sediment. Animals were fixed in formaldehyde solution (5%) prepared with seawater and buffered with sodium borate to prevent the dissolution of calcified structures and facilitate faunal identification. Macrofauna were sorted under a dissecting microscope (10× magnification) and transferred to 70% ethanol. Most specimens were identified to species, except for polychaetes and crustaceans, which were identified to morphospecies and family respectively, and nemerteans and sipunculids, which were grouped by phylum. Use of a mixed taxonomic resolution was necessary because many of Australia’s invertebrate fauna remain undescribed and poorly known. This approach does not compromise the detection of spatial patterns of macroinvertebrates [36], [37].

To assess how the relationship between nutrient enrichment and macrofaunal communities is influenced by the local environmental context, we quantified sediment organic content and grain size. To assess sediment organic content, a subsample of about 4 g was taken from each of the four small sediment samples after homogenization. Coarse woody debris and shell fragments, where found, were excluded prior to analyses. Consequently, samples contained sediment mixed with organic matter. The subsamples were dried at 105°C for 48 h and weighed prior to combustion at 550°C for 4 h. The organic content was calculated as the percentage difference in weight from before to after combustion. Sediment grain size was determined for two randomly selected replicates at each site. The silt/clay fraction was determined by wet sieving through a 63 µm screen. The remaining sediment was dry sieved through a stack of sieves of decreasing mesh size (2000, 1000, 500, 250 and 125 µm) and the weight of each fraction was measured. Median grain size and sorting were calculated using the software GRADISTAT 4.0 [38].

### 3. Statistical Analysis

To test the hypotheses that nutrient enrichment would (1) be correlated to macrofaunal community structure and (2) the strength of this relationship would be determined by the climatic and local environmental context, we collated a matrix of environmental variables. This included: 1) site averages of environmental data collected during this study (sediment organic matter and silt/clay content, median grain size and sorting), and 2) estuary physical and chemical attributes (see Table 1 for complete list) from Roper et al. [33]. The distribution of each environmental variable across sites was visually inspected and an appropriate transformation was applied to minimise skewness. Environmental variables were normalized and principal component analysis (PCA) was used to outline and visualise the relationships between variables.

To assess the contribution of the environmental variables to the variation observed in the macrofaunal community structure we carried out a multivariate regression using distance-based redundancy analysis (db-RDA; [39]). Multivariate multiple regression (DistLM routine) tested the significance of these contributions by fitting a linear model based on Bray-Curtis dissimilarities from log(x +1) transformed abundance data using permutations. Abundances of macrofaunal taxa were summed across the seven replicate cores per site such that sites became replicates. First, we assessed the contribution of each environmental variable to the variation in the macrofauna community structure. Then we used AICc selection criteria [40] and the BEST procedure (PRIMER; [36]) to find a reduced model that retained only variables with good explanatory power. The reduced model was visualized with a db-RDA plot. In order to identify which taxa showed the highest correlation to the set of environmental variables (multiple correlation coefficient >0.3), we superimposed vectors. Using the DistLM routine, we also tested for significant relationships between single discriminating taxa and each environmental variable. Euclidian distance was used as the basis for the analysis and *p*-values were obtained by permutation. In addition, Pearson’s correlation coefficient was calculated for each significant taxon-environmental variable pair.

All multivariate and univariate procedures were carried out with PRIMER v6 [36] and PERMANOVA+ [41] statistical package.

## Results

### 1. Environmental Variables

**Figure 2:**
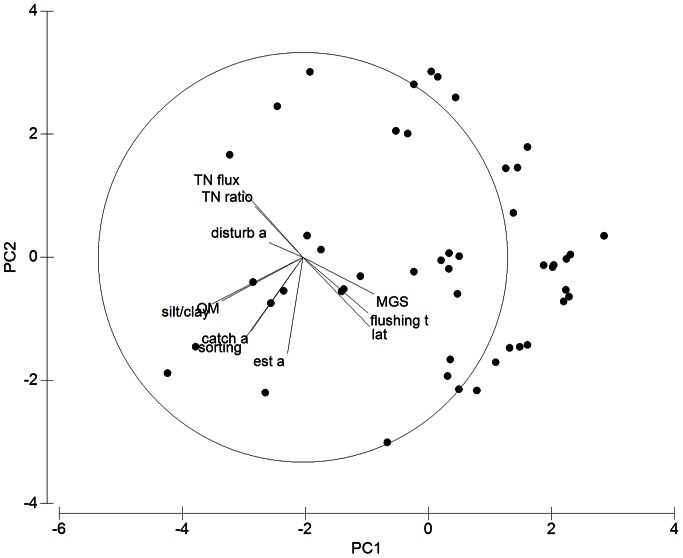
PCA (Principal component analysis) of the 11 environmental variables, excluding longitude, listed in Table 1 (transformed and normalised). Dots represent sites within estuaries. Vectors show the two-dimensional (PC1 and PC2) correlation structure among the environmental variables (% of variance explained = 52.4). Abbreviations: catch a = catchment area, disturb a = % of disturbed area of the catchment, est a = estuary area, flushing t = flushing time, lat = latitude, MGS = median sediment grain size, sorting = sediment sorting, OM = sediment organic matter, silt/clay = % sediment silt/clay content, TN flux = flux of total nitrogen, TN ratio = ratio of total nitrogen loading pre-European settlement to present.

Principal component analysis revealed a high degree of interrelatedness among environmental variables. As a result, the first two principal component axes explained approximately 52% of the total variation. The two-dimensional PCA plot revealed two groups of interrelated variables (Fig. 2). Latitude, flushing time and median grain size (MGS) were negatively correlated with total nitrogen (TN) flux, TN ratio and the percentage of disturbed catchment area. The second group, roughly orthogonal to the first, consisted of sediment silt/clay and organic matter content, sediment sorting and catchment and estuary area.

### 2. Macrofauna

**Figure 3:**
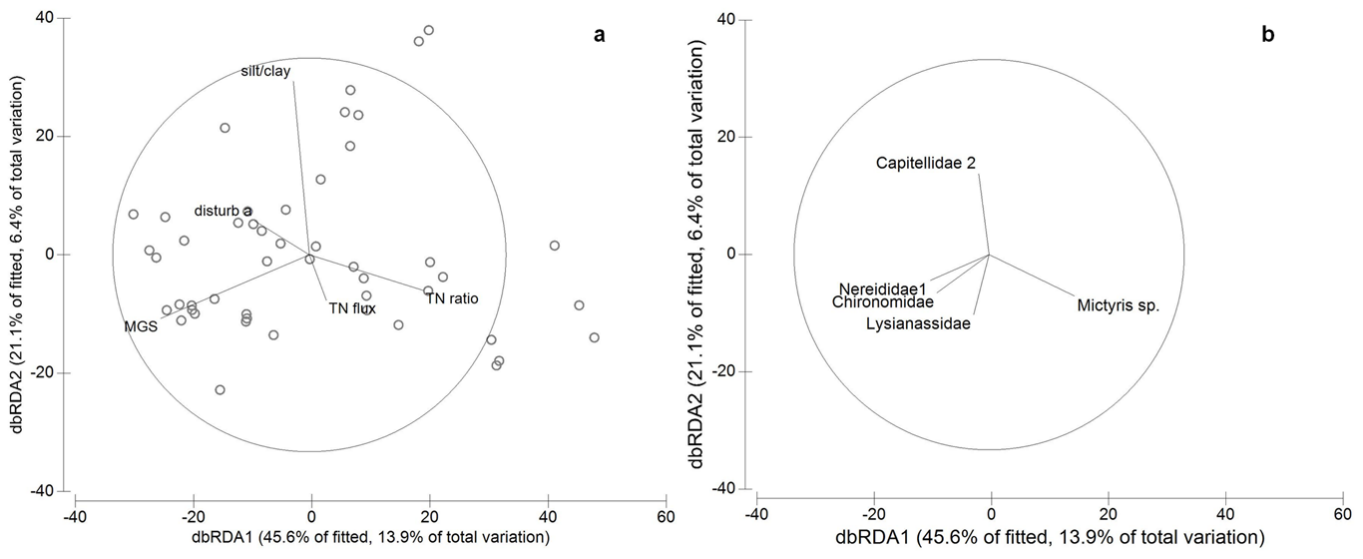
dbRDA plots representing the reduced model of spatial variation in macrofaunal community structure and its relationship to a) environmental variables and b) the abundance of key taxa significantly correlated with db-RDA axes (multiple correlation >0.30). Points omitted in plot b for clarity. See Fig. 2 for abbreviations.

A total of 70 taxa and 18510 macrofaunal individuals were found. Across all estuaries, the mean (± SE) abundance of macrofauna per site (i.e. 7 sediment cores) was 397±67 and the species richness was 13±1. The most abundant group was the polychaete worms, followed by bivalves, chironomid larvae, crustaceans and gastropods. Macrofaunal community structure was weakly correlated with individual environmental variables (Table 2a). Those individual abiotic variables most strongly correlated to macrofaunal assemblages were latitude, median sediment grain size (MGS), flushing time and sediment silt/clay content (Table 2a; Fig. 3). The combination of environmental variables that was most closely correlated to the macrofaunal data, explaining 30% of the variability, included the five variables, sediment MGS and silt/clay, TN ratio, TN flux and % disturbed area (BEST procedure PRIMER, Table 2a; Fig. 3a). *Mictyris* spp. (*M. longicarpus* and *M. platychelis*) soldier crabs, capitellid and nereid polychaetes, chironomids and lysianassids were the taxa that mostly correlated with the multivariate abiotic data (Fig. 3b). When analysed singularly, however, correlations between these species and individual environmental variables were generally weak (Table 3a). The strongest correlations were the negative relationship between *Mictyris* spp. and latitude and the positive relationship between chironomids and each of the variables latitude and MGS, although MGS and latitude were themselves correlated (Table 3a). Only soldier crabs *Mictyris* spp. and chironomids were correlated with TN ratio, and these correlations were positive for *Mictyris* spp. and negative for chironomids (Table 3).

**Table 2:** Results of multivariate multiple regression analyses (distLM) using data from: (a) all estuaries, (b) estuaries with a median sediment grain size of <250 µm and (c) estuaries with a median grain size of >250 µm.

	a) All estuaries			
Environmental variables	Pseudo-F	*p*_perm_	Prop.	Prop. BEST
Latitude	5.31	**0.000**	0.10	
Sediment median grain size	5.28	**0.000**	0.10	0.10
Flushing time^a^	3.49	**0.001**	0.07	
Sediment Silt/Clay^b^	3.07	**0.002**	0.06	0.05
Sediment OM^b^	2.46	**0.009**	0.05	
TN ratio^b^	2.40	**0.010**	0.05	0.07
Sediment sorting^b^	2.22	0.015	0.05	
TN flux^c^	1.99	0.030	0.04	0.05
Catchment area^b^	1.56	0.102	0.03	
Disturbed area	1.54	0.106	0.03	0.03
Estuary area^b^	1.24	0.233	0.03	
	*R^2^ BEST solution*			0.30
	**b) Low median grain size**			
**Environmental variables**	**Pseudo-F**	***p*** **_perm_**	**Prop.**	**Prop. BEST**
Sediment Silt/Clay^b^	3.3	**0.001**	0.13	0.13
Sediment OM^b^	2.8	**0.002**	0.11	
Sediment sorting^b^	2.2	**0.018**	0.09	
Catchment area^b^	2.1	**0.021**	0.09	0.09
Flushing time^a^	2.0	**0.029**	0.08	0.10
Estuary area^b^	1.8	**0.048**	0.08	
TN ratio^b^	1.3	0.216	0.06	
TN flux^c^	1.2	0.269	0.05	
Disturbed area	1.2	0.293	0.05	
Latitude	1.2	0.293	0.05	
Sediment median grain size	1.1	0.342	0.05	
	*R^2^ BEST solution*			0.32
	**c) High median grain size**			
**Environmental variables**	**Pseudo-F**	***p*** **_perm_**	**Prop.**	**Prop. BEST**
Latitude	4.7	**0.000**	0.18	0.18
Sediment sorting^b^	4.5	**0.000**	0.17	
Sediment OM^b^	3.8	**0.000**	0.15	
Sediment Silt/Clay^b^	3.8	**0.000**	0.15	0.09
Flushing time^a^	3.5	**0.000**	0.14	
Catchment area^b^	3.2	**0.001**	0.13	0.12
TN flux^c^	2.8	**0.005**	0.11	
Estuary area^b^	2.8	**0.004**	0.11	
TN ratio^b^	2.7	**0.004**	0.11	0.13
Disturbed area	2.3	**0.016**	0.09	0.10
Sediment median grain size	1.9	0.052	0.08	0.07
	*R^2^ BEST solution*			0.69

Prop. = the proportion of variance in the macrofaunal community structure explained by each environmental variable. Prop. BEST = the proportion of variance explained by the variables selected as the key environmental drivers using the BEST procedure (AICc selection criterion). Significant (*p*_perm_ <0.05) predictor variables are in bold. Macrofaunal data were log(x +1) transformed prior to analysis. Abbreviations: TN flux = flux of total nitrogen, TN ratio = ratio of total nitrogen loading pre-European settlement to present. In order to achieve approximate normally distribution, data were (^a^) square root, (^b^) log and (^c^) 4^th^ root transformed.

**Table 3:** Pearson’s correlation coefficients of significant (*p*_perm_ <0.05, DistLM routine) correlations between the abundances of macrofaunal taxa that were most correlated to abiotic data and individual predictor environmental variables for (a) all estuaries, (b) estuaries with a median sediment grain size of <250 µm and (c) estuaries with a median grain size of >250 µm.

Taxon	Catchment area	% OM	Latitude	MGS	% Silt/clay	Sediment sorting	TNratio	Flushingtime	Estuaryarea	ActualTN flux	Disturb area
**a) All estuaries**											
*Mictyris* spp.	−0.25		−0.54	0.49		−0.31	0.49				
Capitellidae 2				−0.04		0.49					
Chironomidae		−0.41	0.52	0.50	−0.39		−0.45				
Nereididae 1		−0.19	0.41	0.49	−0.21			0.17			0.43
Lysianassidae		−0.28	0.30	0.36	−0.37						
**b) Low median grain size**											
Nereidadae 3	−0.43	0.56			0.46			0.66			
*Casco* sp.								0.47			
Arthritica helmsi	0.54								0.44		
Capitellidae 2		0.47			0.63	0.49	−0.42				
*Capitella* sp.				0.49							
*Mictyris* spp.	−0.40	−0.53	−0.43		−0.59	−0.41	0.49				
*Hydrobia buccinoides*	0.49								0.49		
**c) High median grain size**											
*Capitella* sp.		−0.45	0.76			−0.48	−0.53	0.50		−0.61	
Nereididae 2	0.14					0.54	−0.03	−0.49		0.14	−0.13
Nereididae 1		−0.34			−0.34				−0.33		0.48
*Soltellina alba*	−0.41	−0.22	−0.07	−0.07		−0.46					
* Arthritica helmsi*	−0.61	−0.37	0.14	0.11							
Orbinidae	0.16					0.43	−0.32			0.19	
Chironomidae	−0.47		0.35		−0.49		−0.67		−0.37	−0.14	

TN ratio = ratio of total nitrogen loading pre-European settlement to present. n = 48 for (a), n = 24 for (b) and (c). Data log(x+1) transformed.

Estuaries followed a bimodal distribution in MGS, with two peaks at about 170 and 330 µm and a median value of 255 µm. Therefore, in order to minimize the correlation between latitude and MGS, we separated the dataset into two groups of eight estuaries, based on their MGS value (low MGS <250 µm, high MGS >250 µm), and ran the same multivariate analyses for each subgroup as for the complete data set. The set of low MGS estuaries included seven of the eight northern-most estuaries, plus one in the south. The set of high MGS estuaries included seven of the eight southern-most estuaries plus one in the north.

**Figure 4:**
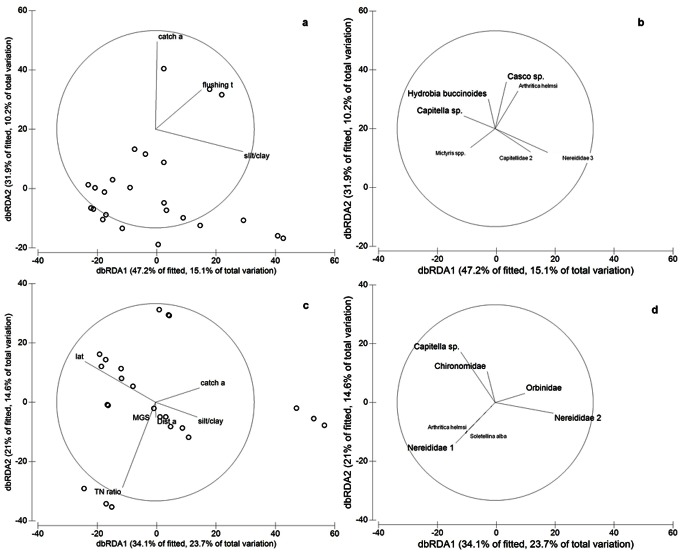
dbRDA plots representing the reduced model of spatial variation in the macrofaunal community structure of estuaries with a median sediment grain size of <250 µm (a, b) and estuaries with a median grain size of >250 µm (c, d). Macrofaunal communities are related to environmental variables (a, c) and taxa significantly correlated with db-RDA axes (b, d) (multiple correlation >0.30). Points omitted in plot b and d for clarity. See Figure 2 for abbreviations.

Across the low MGS ( = lower latitude) estuaries, the mean (± SE) abundance of macrofauna per site (i.e. 7 sediment cores) was 112±25 and the species richness was 12±1. Multiple regression analysis on low MGS estuaries indicated that sediment variables other than MGS explained most of the variability in macrobenthic assemblages (Table 2b). These were silt/clay content, organic matter content, and sediment sorting. Several other physical variables (catchment area, flushing time, estuary area) were more weakly but significantly correlated with macrofaunal community structure (Table 2b). Conversely, TN ratio and flux did not correlate with the macrobenthic assemblage. The combination of environmental variables best explaining overall variation (as indicted by BEST analysis, PRIMER), silt/clay content, catchment area, and flushing time, explained 32% of the variability (Table 2b; Fig. 4a). Nereid and capitellid polychaetes, the amphipod *Casco* sp., soldier crabs *Mictyris* spp. and the gastropod *Hydrobia buccinoides* were the taxa that most closely correlated with the environmental variables of the BEST model (Fig. 4b). Correlations between individual taxa and environmental variables were weak (Table 3b). Nereididae 3 abundance was positively correlated with estuary flushing time and sediment organic matter (Table 3b). Capitellidae 2 was positively and *Mictyris* spp. were negatively correlated with sediment silt/clay content (Table 3b). The abundance of these two species was also significantly correlated with TN ratio, negatively in the instance of Capitellidae 2 and positively in the case of *Mictyris* spp. (Table 3b).

Across the high MGS ( = higher latitude) estuaries, the mean (± SE) abundance of macrofauna per site (i.e. 7 sediment cores) was 682±101 and the species richness was 15±1. Macrofaunal assemblage structure was significantly correlated with each of the environmental variables included in the model, except MGS (Table 2c). Latitude was the individual variable most strongly correlated to macrofaunal communities, explaining 18% of the variability. TN flux and TN ratio were also significantly correlated to the macrofaunal assemblage and each explained 11% of the variability. Sediment silt/clay content, catchment area, disturbance area and MGS, along with latitude and TN ratio were among the group of variables chosen by the BEST procedure as being most correlated to the fauna (Table 2c). Together, the sub-group explained 69% of the total variation (Table 2c; Fig. 4c).

Among the high MGS estuaries, spatial variation in macrofaunal assemblages were driven by variation in *Capitella* sp. and nereid polychaetes, chironomid larvae and the bivalves *Arthritica helmsi* and *Soletellina alba* (Fig. 4d). Analyses of the abundance of discriminant taxa in high MGS estuaries showed that the abundance of *Capitella* sp. was positively correlated with latitude and negatively with TN flux and TN ratio (Table 3c). Other relevant relationships included the negative correlation between chironomid abundance and TN ratio and between *Arthritica helmsi* and catchment area (Table 3c). In addition, TN ratio and flux yielded higher *R* values and stronger correlation with a higher number of taxa compared to low MGS estuaries (Table 3c).

## Discussion

Of the site-specific and estuary-scale environmental variables considered by this study, we found that sediment grain size was the best predictor of macrofaunal assemblage composition. Only when samples were stratified according to median grain size did the correlation between macrofauna and nitrogen loading of estuaries emerge, and even then, only among sites with sandy sediments. Among sites with muddy sediments, the percent contribution of silts and clays to sediment weight and organic content were better predictors of macrofaunal community structure than nutrient loading.

The relationship across all 48 sites sampled between sediment grain size and fauna was consistent with a rich literature on broad-scale differences in macrofaunal assemblages between sandy and muddy sediments [42], [43], [44]. Further, when we separately analysed data from estuaries of high and low median grain size, the organic content and percent by weight of silts and clays remained important correlates of community structure among the low median grain size group. The taxa that were more strongly correlated to sediment characteristics were several species of Nereididae and Capitellidae polychaetes. Generally, these deposit feeding taxa were positively correlated with sediment organic and silt/clay content, as predicted by the paradigm of sediment ecology [45], that posits that deposit feeders will be more abundant in muddy and suspension feeders in sandy sediments.

Nevertheless, whether the relationship between fauna and sediments represented a cause-effect or indirect relationship is unclear, due to the correlative approach of our study. Flow regimes and sediment processing by fauna can determine grain size, but each of these factors may themselves directly influence macrofauna so manipulative experiments are required to disentangle cause-effect relationships [46]. In the present study, the estuaries containing muddier sediments were generally situated towards the northern end of the latitudinal range sampled, while the sandier estuaries, towards the south. As the estuaries to the north, which are situated in a sub-tropical climate, generally receive a greater riverine influence than the temperate estuaries to the south [21], [47], this pattern may alternatively be explained by differences in flow regime [12], [48].

The observed pattern of a stronger relationship between nutrient enrichment and macrofaunal communities in sandy than muddy sediments is consistent with previous research done on a smaller scale, where climate setting was not a confounding variable. In a comparison of the structure of macrofaunal communities between sections of a south-eastern Australian estuary that drained urbanized and forested land, Lindegarth and Hoskin [49] found that impacts of coastal development were confined to sites with sandy sediments. Among sites with muddy sediment, there was no relationship between adjacent land use and macrofaunal assemblage structure [49]. Our study, which sampled across the larger scale of estuaries, rather than sites within an estuary, found a similar pattern at the community-scale. Nevertheless, the specific taxa responding most strongly to disturbance differed between the two studies [49]. This suggests that this pattern is not the result of a set of specific taxa, but, rather, a generalised response of macrofauna living in sandy sediments [49].

Differences in the effect of TN loading on macrofaunal communities between estuaries with sandy and muddy sediments may have arisen in at least three different ways. First, it is possible that macrofaunal taxa responded indirectly to sediment properties because these were correlated with hydrological characteristics (e.g. flushing time, tidal inundation) that would affect the fate and utilization of N in the system [21]. Second, the differing response of macrofauna to nutrient disturbance between the two sediment types may have arisen from intrinsic differences between sandy and muddy sediments in sediment organic content (e.g. [50]). Among muddy sediments, the observed relationship between organic content and macrofaunal communities may have overridden effects of nutrient enrichment. By contrast, the lower background levels of organic matter in the sandier sediments might have allowed for a greater nutrient effect. Third, differences in the assemblage composition of sandy and muddy sediments may be responsible for the differing relationships between nutrient enrichment and macrofaunal community composition between these habitat types [49]. Crustaceans were more abundant in sandy than muddy sediments and chironomid larvae showed the opposite pattern. Furthermore, overall sandy sediments contained more taxa than muddy sediments. Crustaceans are regarded as being very sensitive to environmental perturbations compared to polychaetes [51], [52], [53]. They did not, however, display a strong relationship to nutrient enrichment in this study, perhaps because their identification to family level prevented the detection of changes in species composition. A higher mean number of species in the sandy sediment means that bias, associated with the greater probability of the community including a sensitive taxon, may have also contributed to in the stronger relationship between macrofaunal communities and TN loading in sediment samples.

In contrast to studies done in North America or Europe [54], [26], the relationship between TN loading and macrofaunal assemblage composition found in this study, even in sandy sediments, was weak. This may be attributed to the relatively low loadings of nitrogen that southeast Australian estuaries receive being insufficient to impact benthic faunal communities [26], [55]. Even the most modified of south-east Australian estuaries contain equal or lower TN loading than the most pristine estuaries in the US [56]. The lack of any relationships between TN ratio and sediment organic matter content regardless of grain size, is in agreement with the oligotrophic nature of Australian estuaries. Studies experimentally fertilizing sediments have found minimal impacts of enrichment on macrofaunal assemblages of south-east Australian estuaries [57], [58].

In conclusion, the relationship between nutrient loading and benthic macrofaunal communities varied among sites according to the grain-size of their sediment and was, on the whole, weak. Instead characteristics of the sediment itself, such as grain size and the amount of silts and clays, were much better predictors of macrofaunal communities. Nevertheless, as human population growth continues to place increasing pressure on southeast Australian estuaries, manipulative studies that can ascertain cause-effect relationships are required to examine when and where nutrient loading will lead to significant changes in estuarine community structure, and how changes in flow regime and sedimentation might influence fauna through effects on sediment properties [59]; [46].
